# de Haas-van Alphen effect of correlated Dirac states in kagome metal Fe_3_Sn_2_

**DOI:** 10.1038/s41467-019-12822-1

**Published:** 2019-10-25

**Authors:** Linda Ye, Mun K. Chan, Ross D. McDonald, David Graf, Mingu Kang, Junwei Liu, Takehito Suzuki, Riccardo Comin, Liang Fu, Joseph G. Checkelsky

**Affiliations:** 1 Department of Physics, Massachusetts Institute of Technology, Cambridge, MA 02139 USA; 2 National High Magnetic Field Laboratory, LANL, Los Alamos, NM 87545 USA; 3 National High Magnetic Field Laboratory, Tallahassee, FL 32310 USA; 4 Department of Physics, Hong Kong University of Science and Technology, Clear Water Bay, Hong Kong, China

**Keywords:** Ferromagnetism, Magnetic properties and materials, Topological insulators

## Abstract

Primarily considered a medium of geometric frustration, there has been a growing recognition of the kagome network as a harbor of lattice-borne topological electronic phases. In this study we report the observation of magnetoquantum de Haas-van Alphen oscillations of the ferromagnetic kagome lattice metal Fe_3_Sn_2_. We observe a pair of quasi-two-dimensional Fermi surfaces arising from bulk massive Dirac states and show that these band areas and effective masses are systematically modulated by the rotation of the ferromagnetic moment. Combined with measurements of Berry curvature induced Hall conductivity, our observations suggest that the ferromagnetic Dirac fermions in Fe_3_Sn_2_ are subject to intrinsic spin-orbit coupling in the *d* electron sector which is likely of Kane-Mele type. Our results provide insights for spintronic manipulation of magnetic topological electronic states and pathways to realizing further highly correlated topological materials from the lattice perspective.

## Abstract (web summary)

The kagome lattice is increasingly known as a host for correlated topological electronic states. Here, Ye et al. report quantum de Haas-van Alphen oscillations of a ferromagnetic kagome material Fe3Sn2, where bulk electronic Dirac fermions are found to be modulated by rotation of the magnetic moment.

## Introduction

The field of topological electronic materials has seen rapid growth in recent years^1,2^, in particular with the increasing number of weakly interacting systems predicted and observed to host topologically nontrivial bands^3–5^. Despite the vast number of materials identified as topological in nature, design principles of electronic topology besides numerical identification involving extensive band details are much less established. This particularly impedes the quest for correlated topological materials^6,7^, where calculation is known to be challenging to yield precise band information. The theoretical development of the topological band theories itself has relied heavily on conceptual lattice models^8^; to what degree such models are relevant for real materials remains an open question.

The two-dimensional (2D) kagome lattice is a system of corner-sharing triangles assembled in a hexagonal fashion analogous to the graphene lattice^9^. These triangular and hexagonal structural features are a test ground to access the physics of magnetic frustration^10^ and of lattice-driven Dirac fermions^11^, respectively. In the context of electronic hopping models, the kagome network also gives rise to a flat band together with a pair of Dirac bands that potentially support exotic phases such as interaction-driven ferromagnetism^12,13^ and chiral superconductivity^14^. In reciprocal space, the two Dirac band touching points on the kagome lattice are positioned at the *K* and *K*′ points at the Brillouin zone boundary, identical to the case for the graphene lattice, and likewise are protected by crystallographic symmetries^9^. Compared with the graphene lattice, the nearest-neighbor bonds in the kagome lattice are not contained in mirror planes perpendicular to the basal lattice, and can therefore experience an electric field orthogonal to the nearest-neighbor bonds^15^, introducing explicitly spin–orbit effects into the band structure and pathways to topologically nontrivial electronic bands^16,17^.

**Fig. 1:**
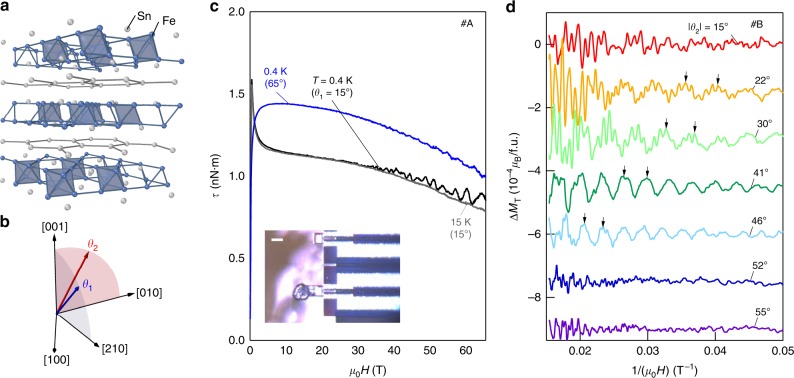
Pulsed field torque magnetometry and de Haas–van Alphen oscillations in Fe_3_Sn_2_. **a** Three-dimensional crystal structure of Fe_3_Sn_2_ showing the Fe kagome bilayers partitioned by stanene honeycomb layers. The blue clusters are defined by the shortest Fe–Fe bonds (<2.55 Å). **b** Depiction of rotation of the magnetic field from out-of-plane to two inequivalent in-plane principal directions. The angles between the field and *c* axis are defined as *θ*_1_ and *θ*_2_ in the two rotation planes, respectively. **c** Magnetic torque *τ* measured up to 65 T for *θ*_1_ = 15° and 65° with de Haas–van Alphen oscillations observed above ~20 T for *T* = 0.4 K. The inset shows an optical image of the piezoresistive cantilever with one crystal of hexagonal, plate-like Fe_3_Sn_2_ (the scale bar is 50 μm). **d** Oscillatory part of the transverse magnetization Δ*M*_T_ at selected angles at base temperature *T* = 0.5–0.6 K versus inverse magnetic field. The black arrows correspond to the eighth and ninth oscillation of the slow frequency at each angle

In terms of material realizations, a number of recent efforts have focused on metallic kagome lattice materials that potentially connect to the electronic hopping behavior expected for the 2D lattice. In particular, the kagome lattice has been realized in a series of hexagonal 3*d* transition metal stannides and germannides^18,19^. The basic building blocks consist of a transition metal kagome layer *T*_3_(Ge,Sn) with Sn/Ge at the hexagon center, together with a stanene layer (Ge,Sn)_2_ (see Fig. 1a), forming compounds with the chemical formula [*T*_3_(Ge,Sn)]_*x*_[(Ge,Sn)_2_]_*y*_. Two representative materials, Mn_3_Sn (*x* = 1, *y* = 0)^20^ and Fe_3_Sn_2_ (*x* = 2, *y* = 1)^21,22^ have recently been identified as hosts to 3D Weyl fermions and quasi-2D massive Dirac fermions, respectively, suggesting that the dimensionality of electronic topology is sensitive to the crystallographic arrangement of the basic building blocks, attributed to covalent and metallic bonding of the stanene and kagome layers, respectively^23^. The importance in this construction can also be seen by comparing with kagome lattice-containing Co_3_Sn_2_S_2_—there, despite a relatively large layer spacing, the electronic structure is 3D in nature as the additional sulfur network bridges the kagome layers^24,25^. The use of the 3*d* transition elements allows the introduction of magnetism; in the case of Fe_3_Sn_2_, this is a soft ferromagnetic order^26^, which along with atomic spin–orbit coupling, gives rise to substantial intrinsic anomalous Hall conductivity from the massive Dirac bands extending above room temperature^21^. Given the softness of this magnetic order, a natural question that arises is how a general positioning of the magnetic moment **m** affects the electronic structure and topology of this system. For Kane–Mele-type spin–orbit coupling, the orthogonality between the local electric field and magnetic moment orientation can selectively open the gap; such control of electronic topology with magnetic moment orientation is uniquely enabled in ferromagnetic systems^27^.

Here we report a torque magnetometry study that captures the evolution of the quasi-2D Dirac bands via the de Haas–van Alphen effect (dHvA) while at the same time monitoring changes in the magnetic order. These observations together demonstrate a systematic development of the massive Dirac states consistent with a Kane–Mele spin–orbit coupling^8^ with a relativistic energy shift comparable to those observed in elemental ferromagnets^28–32^.

## Results

### de Haas–van Alphen effect in Fe_3_Sn_2_

Measurement of the magnetic torque *τ* for Fe_3_Sn_2_ up to 65 T for two different angles *θ*_1_ = 15° and 60° (see Fig. 1b) is shown in Fig. 1c for the applied field *H* relative to the *c* axis of the crystal. For both angles, an initial rise with *H* gives way to a gradual decay, while a sharp low-field peak emerges for *θ*_1_ = 15°. As we return to below, this corresponds to the polarizing process of the soft ferromagnetic moment along the field direction, with **m** being aligned along *H* with a deviation <0.02 *µ*_B_/f.u. above 10 T. For temperature *T* = 0.4 K, as *H* increases above ~20 T, we see the onset of dHvA oscillations. As shown for *θ*_1_ = 15°, at higher *T* = 15 K, the oscillations are suppressed, while the overall shape of *τ*(*H*) remains relatively unchanged. This is indicative of the lower energy scale for Landau quantization compared with the magnetic order and the associated anisotropy (see Supplementary Note 1). Figure 1d shows the oscillatory component in the transverse magnetization ${\mathrm{\Delta }}M_{\mathrm{T}} \equiv {\mathrm{\Delta }}\tau /\mu _0H$ (Δ*τ* is the oscillatory part of torque after subtracting a polynomial background) as a function of inverse applied field Δ*M*_T_(*H*^–1^) at *T* = 0.5 K for various *θ*_2_. Multiple frequencies are evident accompanied by an increase in frequency of the slowest oscillation with increasing *θ*_2_ (black arrows in Fig. 1d trace its eighth and ninth Landau level). The magnitude of the oscillations is consistent with a bulk origin (surface-state oscillations would correspond to an amplitude of ~4 *µ*_B_ per surface unit cell, comparable with ***m*** itself^33^).

**Fig. 2:**
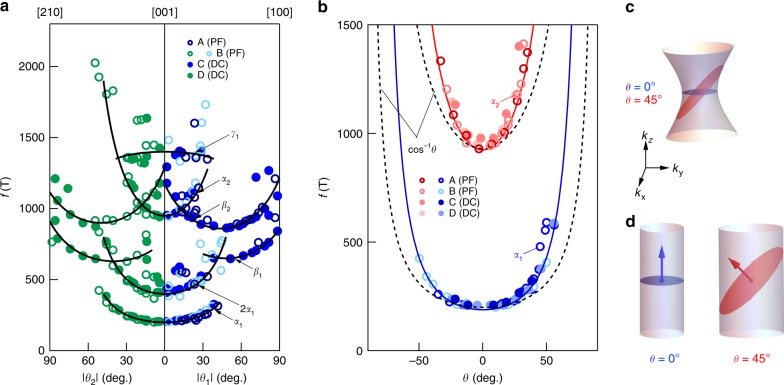
Angular dependence of dHvA oscillation frequencies. **a** Angular dependence of all fast Fourier transform (FFT) frequencies with rotation from [001] to [010] (left panel) and from [001] to [210] direction (right panel). Empty circles are collected from pulsed field experiments, while solid circles are from DC field experiments. Data taken from different samples are represented with different colors. The black curves are guides to the eye. **b** Angular dependence of *α*_1_ and *α*_2_ pockets. The dashed lines are the behavior expected for a 2D cylindrical Fermi surface (1/cos *θ*), and the solid lines are a massive Dirac model (see text). **c** Schematic of a hyperboloid Fermi surface whose smallest extremal area evolves faster than 1/cos *θ* with a rotating magnetic field. **d** Schematic of quasi-2D Fermi surface where the *k*_*z*_-dispersionless Fermi wave vector changes with the direction of magnetization (shown as arrows)

The dHvA spectrum for samples A–D at the base *T* = 0.5–0.6 K for oscillation frequency *f* determined from a fast Fourier transform (FFT) of the oscillatory torque Δ*τ* is shown in Fig. 2a (for a full angular spectrum see Supplementary Fig. 4). Samples A and B (empty circles) were measured with pulsed fields up to 65 T, while C and D (solid circles) were measured in DC fields up to 35 T. The responses with *H* rotated from [001] to [210] (*θ*_1_ rotation) and from [001] to [010] (*θ*_2_ rotation) are similar as shown in Supplementary Fig. 5 (hereafter we refer to both as *θ*); we identify five branches in the oscillatory pattern with the qualitative behaviors we label as *α*_*i*_, *β*_*i*_ (*i* = 1,2) and *γ* where the harmonic of *α*_1_ is also observed. The *α* group grows rapidly with *θ* toward a divergence as *H* approaches the basal plane, implying that they have a quasi-2D nature. The value of *f*(*θ*) approaching the *c* axis *f*_0_ for *α*_1_(*α*_2_) is ~200 T (930 T), corresponding to a Fermi wave vector $k_{\mathrm{F}} \approx 0.08 {\,} {\it{{\AA}}}^{ - 1}( {0.17{\,}{\it{{\AA}}}^{ - 1}})$ similar to the inner (outer) Dirac cone areas observed in angle-resolved photoemission spectroscopy (ARPES) at *K* and *K*′^21^. We therefore identify these *α* bands as the quasi-2D massive Dirac bands derived from the Fe kagome network with wave functions primarily confined to the plane and confirm their bulk nature. In contrast, the *β* and *γ* groups are free from divergences and instead follow angular dependencies suggestive of three-dimensional, closed Fermi sheets, which we identify with the *k*_*z*_-dispersive bands previously reported^21^.

### Moment orientation–dependence of massive Dirac fermions

We examine the *α* bands in more detail in Fig. 2b. For an ideal 2D Fermi surface, an evolution $f(\theta ) = f_0/\cos \theta$ is expected. This dependence is shown as a dashed line in Fig. 2b—we find that the evolution of both of *α* bands increases more rapidly than this dependence. A deviation of this type is observed in systems with local hyperboloid geometries as depicted in Fig. 2c^28,34^. However, this is at odds with the lack of *k*_*z*_ dispersion in ARPES^21^. Moreover, a sinusoidal *k*_*z*_ warping accommodating such a geometry is unable to capture the observed $f\left( \theta \right)$, and moreover predicts counterpart extremal frequencies and Yamaji angles (41° and 69°) that are absent^35^ (see Supplementary Fig. 9). Interestingly, similar apparently contradictory dHvA spectra were previously observed in elemental ferromagnets^28,30,31^, where it was eventually realized for Ni^30^ that this could be resolved by considering that spin–orbit coupling would introduce a shift of the energy of the elliptical Fermi pockets up to 50 meV depending on the magnetization direction. Applying such a scenario to the present case of a quasi-2D surface is shown in Fig. 2d, where *H* plays a dual role setting the direction of magnetization and the plane for cyclotron motion, introducing a faster-than- $1/\cos \theta$ development. As we describe below, this is well described by a massive Dirac model with systematically evolving band parameters constructed for the inner Dirac band and extended to capture the outer Dirac band (solid lines in Fig. 2b).

We note from these observations that the size of the Fermi surface can be used as a caliper to probe the orientation of **m**. By focusing on the smaller Dirac surface, from ARPES performed between 90 and 110 eV, a *k*_*z*_-independent Fermi wave vector is observed corresponding to a circular Fermi surface area $A_k = \left( {0.0259 \pm 0.0008} \right)\,{\it{{\AA}}}^{ - 2}$. By converting this to an angular projected area, we find that it corresponds to the *c*-normal Fermi surface in Fig. 2b at $\theta = \left( {43 \pm 2} \right)^{\mathrm{o}}$ or a *z* component of the magnetic moment $m_z \approx 0.7\left| {\mathbf{m}} \right|$. Studies of bulk magnetic order in Fe_3_Sn_2_ have reported a spin reorientation of the moments toward the basal plane with varying degrees of *c*-axis moment at *T* = 20 K (at which ARPES was performed) accompanied by a variety of magnetic orders including collinear^36^, non-collinear^26^, and spin glass^37^, while surface probes have suggested that such a reorientation may be first order and partial or complete depending on cooling history^37^. The acute dependence of $f\left( \theta \right)$ to the ferromagnetic order observed here offers a unique window to map the orientation of **m** for comparison with other surface or bulk-sensitive experiments.

**Fig. 3:**
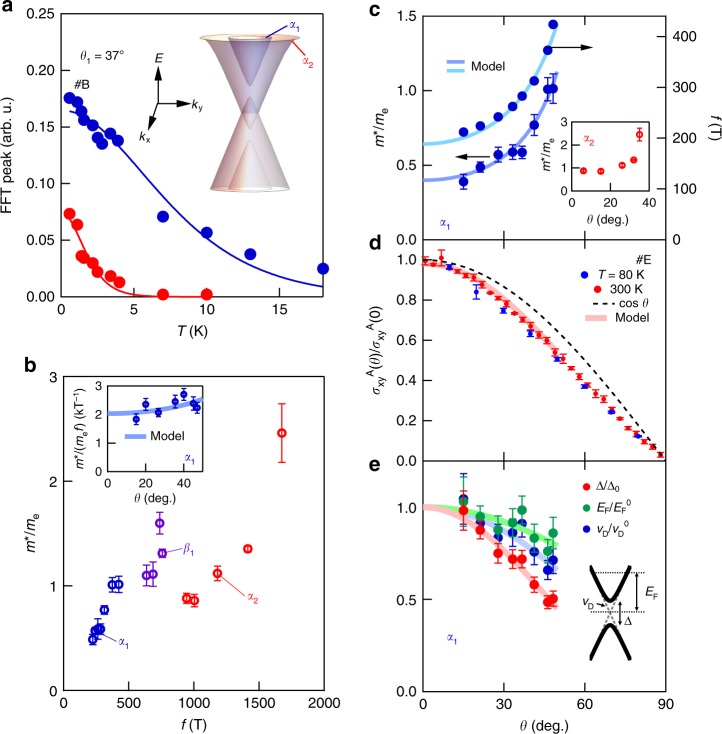
Massive Dirac model of de Haas–van Alphen effect in Fe_3_Sn_2_. **a** Temperature dependence of oscillation amplitude and Lifshitz–Kosevich fitting of *f* = 1675 T (*α*_2_) and 283 T (*α*_1_) at *θ*_1_ = 37°. The inset shows a schematic of the double Dirac spectrum. **b** The observed effective mass *m*^∗^/*m*_e_ versus oscillation frequency *f* for observed Fermi pockets. The inset shows the angular dependence of the ratio *m*^∗^/*m*_e_*f* for *α*_1_ along with the massive Dirac model (see text). **c** Angular dependence of *m*^∗^/*m*_e_ and *f* for the inner Dirac pocket (outer *m*^∗^/*m*_e_ pocket shown in the inset), and **d** anomalous Hall conductivity $\sigma _{xy}^{\mathrm{A}}$ normalized to the zero-angle value ($\sigma _{xy}^{\mathrm{A}}\left( 0 \right) = 130 \, {\mathrm{\Omega }}^{ - 1}{\mathrm{cm}}^{ - 1}$ at 300 K and $\sigma _{xy}^{\mathrm{A}}\left( 0 \right) = 169 \, {\mathrm{\Omega }}^{ - 1}{\mathrm{cm}}^{ - 1}$ at 80 K), respectively, with solid curves showing the massive Dirac model (see text). **e** Angular dependence of the massive Dirac band parameters where the gap is normalized to ${\mathrm{\Delta }}_0 = 32\,{\mathrm{meV}}$, the Dirac velocity normalized to $v_{\mathrm{D}}^0 = 2.2 \times 10^5\,{\mathrm{m}} \cdot {\mathrm{s}}^{ - 1}$, and the Fermi energy is normalized to $E_{\mathrm{F}}^0 = 112\,{\mathrm{meV}}$ with a schematic Dirac band shown in the inset. Error bars correspond to standard errors in least-squares fitting

To further examine the orientational effect of **m** on the electronic structure, we have measured the effective mass *m*^∗^ of the Dirac bands as a function of *θ*. A typical measurement of the dHvA oscillation amplitude (*θ* = 37°) as a function of *T* for both *α* bands is shown in Fig. 3a with the double Dirac structure shown as the inset. The overall dHvA oscillation amplitude of multiple oscillations in magnetization can be written as^34^

1 $$M_{{\mathrm{osc}}} = \mathop {\sum }\limits_i A_iB^{1/2}R_{\mathrm{T}}^iR_{\mathrm{D}}^iR_{\mathrm{S}}^i{\mathrm{cos}}\left[ {2\pi \left( {\frac{{f_i}}{B} + \gamma _i} \right)} \right]$$

where *i* is the band index, *A*_*i*_, *f*_*i*_, and *γ*_*i*_ are the initial amplitudes, oscillation frequencies, and phase factor of the *i*th band, respectively. $R_{\mathrm{T}}^i = \frac{{2{\rm{\pi }}^2k_{\mathrm{B}}Tm_i^ \ast }}{{\hbar eB}}{\mathrm{sinh}}^{ - 1}\left( {\frac{{2{\rm{\pi }}^2k_{\mathrm{B}}Tm_i^ \ast }}{{\hbar eB}}} \right)$ represents the thermal damping factor, $R_{\mathrm{D}}^i = {\mathrm{exp}}\left[ { - \frac{{2{\rm{\pi }}^2k_{\mathrm{B}}T_{\mathrm{D}}^im_i^ \ast }}{{\hbar eB}}} \right]$ is the Dingle damping factor induced by residual impurities where *T*_D_ is the Dingle temperature, *k*_B_ the Boltzmann constant, and 2*π*$\hbar$ the Planck constant. $R_{\mathrm{s}}^i$ is the modulation due to interfering up and down spin oscillations with spin splitting induced by the magnetic field taken here to be unity given the ferromagnetic spin splitting in excess of 1 eV^21^. By fitting $R_T^i$ and using the mean inverse field of the FFT window $\bar B^{ - 1} = \frac{1}{2}\left( {B_{{\mathrm{min}}}^{ - 1} + B_{{\mathrm{max}}}^{ - 1}} \right)$ $\left( {\bar B = 30\,{\mathrm{T}}} \right)$, we find that the *α*_1_ oscillation has effective mass of (0.59 ± 0.04) *m*_e_, while the *α*_2_ pocket has a mass of (2.5 ± 0.3) *m*_e._

By extending this analysis across the dHvA spectrum (see Supplementary Fig. 7), we plot the observed effective masses versus *f* in Fig. 3b. We see a monotonic increase in *m*^∗^ with *f*, but interestingly the ratio *m*^∗^/*f* for *α*_1_ is weakly dependent on *θ* (see Fig. 3b inset). As rigid ellipsoidal, hyperboloid^28^, or quasi-2D pockets^38^ would have a constant ratio, this further suggests the use of a model with a Fermi surface that itself evolves with *θ*. Based on previous observations of the double massive Dirac spectrum in this system (see schematic in Fig. 3a), we analyze the dHvA spectrum with a massive Dirac model. We note that the outer Dirac pocket has been observed to have substantial warping near the Fermi level *E*_F_ (illustrated in the inset in Fig. 3a and observed with the rapidly growing *m*^∗^(*θ*) shown in Fig. 3c inset) and overlaps with other frequencies; we focus the model on the inner Dirac pocket and approximate the outer Dirac pocket as a copy of this band shifted by the observed $E_{\mathrm{\Delta }} = 110$ meV^21^, taken to be fixed here. In analogy to the spin–orbit models of Ni^30^, we consider that the Dirac band parameters are modulated by **m**. We take the Fermi level (defined from the Dirac point) to be $E_{\mathrm{F}} = \sqrt {( {\hbar v_{\mathrm{D}}k_{\mathrm{F}}} )^2 + ( {\Delta /2} )^2}$, where *ν*_D_ is the Dirac velocity, *k*_F_ the Fermi wave vector, and *Δ* the Dirac gap. We can then express *f* and *m*^∗^ (shown in Fig. 3(c)) as

2 $$f = \frac{{E_{\mathrm{F}}^2 - \left( {\Delta /2} \right)^2}}{{2e\hbar v_{\mathrm{D}}^2\cos \theta }},\,m^ \ast = \frac{{E_{\mathrm{F}}}}{{v_{\mathrm{D}}^2\cos \theta }}$$

where *Δ*, *E*_F_, and *ν*_D_ are *θ*-dependent band parameters (cos *θ* is the geometric factor associated with the tilted magnetic field, see also the “Methods” section). In the Δ → 0 limit, such models have been previously applied to graphene to successfully describe the disappearing cyclotron mass at charge neutrality^39,40^. By assuming a Kane–Mele spin–orbit coupling with massive Dirac fermions^8^, the intrinsic anomalous Hall conductivity per kagome bilayer provides a further constraint to these parameters

3 $$\sigma _{xy}^{\mathrm{A}}t = \frac{{e^2}}{{2h}}\left( {\frac{\Delta }{{E_{\mathrm{F}}}} + \frac{\Delta }{{E_{\mathrm{F}} + E_\Delta }}} \right)$$

Here *t* is the thickness of a structural unit that contains a single kagome bilayer. The room-temperature $\sigma _{xy}^{\mathrm{A}}$ (Fig. 3d) is dominated by the Berry-curvature-induced response and is nearly *T* independent from 2 to 400 K^8^; we use this along with *f* and *m*^∗^ to quantify the three independent band parameters within this simplified model.

We show the directly calculated Δ, *E*_F_, and ν_F_ in Fig. 3e along with a schematic band model inset (note: these are calculated from the experimental observations and are not the results of fitting). Generally, all three parameters are suppressed with increasing *θ* that can be reasonably captured by polynomials in cosine of the form *A*(*θ*) = *A*_0_ + *A*_1_ cos *θ* + *A*_2_ cos^2^ *θ* (*A*_*i*_ > 0). With these smooth functions we obtain the solid fits to Fig. 3c, d. In the *θ* → 0 limit, we estimate Δ_0_ = 32 meV, $v_{\mathrm{D}}^0 = 2.2 \times 10^5{\,}{\mathrm{m}} \cdot {\mathrm{s}}^{ - 1}$, and $E_{\mathrm{F}}^0 = 112\,{\mathrm{meV}}$ for the dispersions with moment along the *c* axis. An extrapolation of the model suggests that for the moment near the basal plane, a total shift in *E*_F_ is ~50 meV of comparable scale to that reported in Ni^30^. Δ shows a stronger reduction, while *ν*_*D*_ decreases by 34% up to 50°, suggesting increased correlation of the Dirac states for moments in the plane. We can use the band parameters to reconstruct the trends observed in experiment for the inner Dirac surface (solid curves in Fig. 2b, Fig. 3b–d); while the outer Dirac surface is beyond our model, we find that a simple scaling of *f* from the inner Dirac surface by a factor of 4.9 approximately captures its angular evolution (see Fig. 2b). We note that for *θ* = 42° we infer Δ_0_ = 18 meV, $v_{\mathrm{D}}^0 = 1.67 \times 10^5\,{\mathrm{m}} \cdot {\mathrm{s}}^{ - 1}$, and $E_{\mathrm{F}}^0 = 95\,{\mathrm{meV}}$, in reasonable agreement with the band parameters observed in ARPES^21^, particularly considering the simplicity of the present model. These observations suggest that in the presence of spin–obit coupling, the Dirac bands have a considerable response to changes in the intrinsic ferromagnetism^22^ where the spin–orbit coupling is likely of Kane–Mele type. The spin–orbit coupling energy scale in the present system is substantially enhanced compared with that in graphene^8^ and provides a model system for studying the topological phases associated with Kane–Mele term. Extending the angular range of these measurements, as well as more sophisticated modeling of this behavior, including the role of the other electronic bands as charge reservoirs, are of considerable interest. While modeling the evolution of the three-dimensional bands is more challenging owing to their angular evolution from intrinsic ellipticity, further theoretical efforts in understanding the electronic structure may help to elucidate the spin–orbit-induced changes exiting therein.

### Ferromagnetic torque response in Fe_3_Sn_2_

**Fig. 4:**
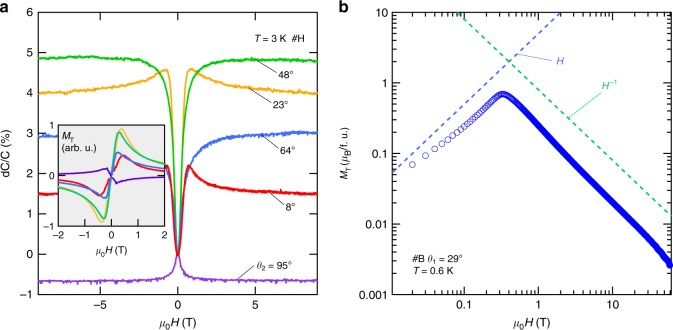
Torque response from the soft ferromagnetism in Fe_3_Sn_2_. **a** Low-field magnetic torque at selected angles at *T* = 3 K measured with a capacitive cantilever in a superconducting magnet. At low angles, the torque response exhibits an initial increase that gradually transforms to a broad shoulder at high angles, consistent with the observation at high fields with piezoresistive cantilevers. The inset shows the transverse magnetization extracted for each torque curve. **b** Pulsed field transverse magnetization *M*_T_ up to 60 T at *θ*_1_ = 29° at *T* = 0.61 K shown in a log–log scale. *M*_T_ attains a maximum ~0.7*μ*_*B*_ per formula unit below 1 T and at higher fields follows an approximately *H*^–1^ dependence

Finally, we return to the overall magnetic torque behavior with *H*. In Fig. 4a we show the low field torque response for different *θ* measured up to 9 T in a superconducting magnet at *T* = 3 K. Similar to the response to high field pulses, for small *θ*, a sharp kink appears followed by a gradual decay, which evolves to a broader shoulder at larger *θ*. We note that the sign changes in the torque response as expected from the change in the quadrant for *θ* = 95°. Despite the apparent qualitative distinction in the torque profiles at small and large *θ*, all the corresponding *M*_T_ curves (Fig. 4a inset) behave similarly, showing an initial sharp growth consistent with the soft ferromagnetic nature^26^ followed by a long tail as a function of field in various angles following primarily $\left( {\mu _0H} \right)^{ - 1}$. The latter corresponds to constant *τ* expected when **m** is effectively saturated along *H*^41^. This trend is clearer when extended to high field: Fig. 4b shows *M*_T_ up to 60 T, showing that it is a good approximation that the moment direction is fixed to the applied field (with deviation < 0.1° or 0.01 *μ*_*B*_/f.u.) above 20 T, thus decoupling the evolution of ***m*** along with the band structure at low fields from the high-field regime in which the dHvA oscillations are observed. Quantitatively, from the angular dependence of the torque, a moderate easy-plane anisotropy can be inferred (see Supplementary Note 1), similar to previous reports in which shape anisotropy plays an important role^42^. Further study of the interplay of bulk, surface, and shape anisotropies with the electronic structure of this system^43^ is an important area for future work; as the Dirac mass itself can influence magnetic order in similar systems^44,45^, an exciting prospect is that the Dirac fermions themselves along with spin–orbit coupling play a role in determining evolution of the magnetic order.

## Discussion

The dHvA results presented here are a thermodynamic probe of the ground state in the presence of a strong polarizing magnetic field of the correlated, topological bands of Fe_3_Sn_2_. The magnetoquantum oscillations confirm the bulk nature of the quasi-2D massive Dirac bands arising from the kagome network previously observed spectroscopically^21^, and provide guidance for theoretical models of this system. Viewed more broadly, the results here demonstrate how lattice-derived topological electronic bands can be wed with the robust ferromagnetism in correlated electron systems. Given the widespread use of 3*d* ferromagnets in spintronics, this provides the exciting prospect that topologically nontrivial analogs of the workhorse materials for spintronics may be developed, allowing direct integration of topological electronic states into such architectures^46,47^. The development of such materials where the charge, spin, and heat transport properties are dominated by the topological bands and controllable with spintronic techniques will be an important direction in realizing the promise of topological electronic states to impact the next generation of electronic devices.

## Methods

### Crystal growth and characterization

Single crystals were grown with an I_2_-catalyzed reaction starting from stoichiometric Fe and Sn powders^21^. The evacuated quartz tube containing the starting materials and I_2_ was placed in a temperature gradient from 750 °C to 650 °C for 3–5 weeks and was quenched in cold water at the end of the growth. Hexagonally shaped crystals were formed near the hot side.

### High magnetic field measurements

Piezo torque magnetometry measurements were performed in the National High Magnetic Field Laboratory (NHMFL) at both the DC field (Tallahassee, Florida) and pulsed field (Los Alamos National Laboratory, LANL) facilities. Measurements in the DC field up to 35 T were performed with PRC-400 (Seiko) cantilevers^48^ in ^3^He atmosphere by using the standard lock-in technique with 50 mV AC excitation voltage (~10–20 Hz) to the bridge circuit. Measurements in the pulsed field up to 65 T were performed by using PRC-120 (Seiko) cantilevers^48^ at LANL in both ^3^He and ^4^He atmospheres with a typical high-frequency (~300 kHz) AC excitation current ~297 μA. We have repeated the measurements and compared the oscillation amplitudes in ^4^He gas at 4 K with different currents to confirm that this measurement current does not induce significant heating. Temperatures between 1.5 and 4 K were taken with the sample immersed in ^4^He liquid.

In both experiments, we used a balanced Wheatstone bridge between the piezoresistive pathways with and without the sample to eliminate contributions from the temperature and magnetic field dependence of the piezoresistor to the torque signal^49^. Crystals were mounted with the *c* axis perpendicular to the cantilever plane and piezo cantilever arm perpendicular (*θ*_1_ rotation) or parallel (*θ*_2_ rotation) to the hexagonal edge. We converted the measured voltage signal to magnetic torque by using the following conversion $\tau = {\mathrm{\Delta }}V/\left( {5.2 \times 10^6V_0} \right) \, {\mathrm{N}} \cdot {\mathrm{m}}$ suggested in ref. ^49^. Here Δ*V* refers to the voltage difference between the two bridge points, and *V*_0_ stands for the excitation voltage to the bridge circuit.

### Capacitive torque measurements

Low-field torque measurements were performed in a commercial superconducting magnet by using 10–25 μm Cu:Be foil, and the signal was acquired with Andeen-Hagerling 2500 AC capacitance bridge. The crystal was attached to the cantilever foil with H20E silver epoxy to prevent detachment in the magnetic field. A typical value of the zero-field capacitance is 0.68 pF at *T* = 3 K in the ^4^He atmosphere.

### Electrical transport measurements

The angular-dependent anomalous Hall effect was measured with the standard five-probe method by using a typical AC excitation current of 2 mA. Both current and voltage leads are placed within the kagome basal plane with current along the [010] direction. The sample was rotated in the magnetic field with *H* approaching from the *c* axis ([001]) to the [210] direction (the angular behaviors observed when *H* is rotated from the *c* axis to the [010] current direction are similar). As the system does not have a remnant magnetization, the anomalous Hall effect was estimated for each angle as the zero-field extrapolation from the linear high-field response.

### Effective mass of Dirac fermions

The effective mass in quantum oscillations is defined proportional to the energy derivative of Fermi surface area with energy $m^{\ast} = \frac{{{\hbar} ^{2}}} {{2{{\pi}}}} \left( {\frac{{{\mathrm{d}} A}}{{{\mathrm{d}} E}}} \right) |_{E_{\mathrm{F}}}$, where *A* is the Fermi surface area and *E* is the energy^34^. For a massive Dirac fermion $E_{\mathrm{F}} = \sqrt {\left( {\frac{\Delta }{2}} \right)^2 \, + \, \left( {\hbar v_{\mathrm{D}}k_{\mathrm{F}}} \right)^2}$, together with $A = {\rm{\pi}} k_{\mathrm{F}}^2$ we can express *A* in terms of *E*: $A = \frac{{\rm{\pi}}} {{\hbar}^{2} v_{\mathrm{D}}^{2}} [ E_{\mathrm{F}}^{2} - \left( \frac{\Delta} {2} \right)^{2} ]$, therefore $\frac{{{\mathrm{d}}A}} {{{\mathrm{d}}E}} = \frac{{2{\rm{\pi}} E_{\mathrm{F}}}} {{{\hbar}^{2} v_{\mathrm{D}}^{2}}}$ that leads to an effective mass $m^ \ast = \frac{{E_{\mathrm{F}}}}{{v_{\mathrm{D}}^2}}$. This formula also applies to the limit where Δ → 0 and has been employed to describe the Fermi level dependence of the effective mass in the quantum oscillation in graphene^39,40^. In our case, we added an additional 1/cos *θ* factor to describe the oblique magnetic field configuration.

## Supplementary information

Supplementary Information

## Data Availability

The data that support the findings of this study are available from the corresponding author on reasonable request.
