# Supplementary material for: de Haas-van Alphen effect of correlated Dirac states in kagome metal Fe3Sn2
Source: Nat Commun. 2019 Oct 25;10:4870. doi: 10.1038/s41467-019-12822-1 (PMC6814717; doi:10.1038/s41467-019-12822-1)
Supplement: Supplementary file 1 — Supplementary Information [file 41467_2019_12822_MOESM1_ESM.pdf]

Supplementary Information

de Haas-van Alphen effect of correlated Dirac states in kagome metal Fe_3Sn_2

Linda Ye[1], Mun K. Chan[2], Ross D. McDonald[2], David Graf[3], Mingu Kang[1], Junwei Liu[4], Takehito Suzuki[1], Riccardo Comin[1], Liang Fu[1], Joseph G. Checkelsky[1]

[1]Department of Physics, Massachusetts Institute of Technology, Cambridge, MA 02139, USA

[2]National High Magnetic Field Laboratory, LANL, Los Alamos NM 87545, USA

[3]National High Magnetic Field Laboratory, Tallahassee, FL 32310, USA

[4]Department of Physics, Hong Kong University of Science and Technology, Clear Water Bay, Hong Kong, China

Supplementary Note 1 | Torque profile in broad angular and temperature range

In Supplementary Fig.1(a) we show the field profile of magnetic torque (τ) up to applied fields of $\mu_0 H = 65$ T at the base temperature (T = 0.45 K ~ 0.6 K) for θ_1 = -85° to 85° for sample A ($\theta_1 = 0$ corresponds to H parallel to the normal of the kagome plane). de Haas-van Alphen (dHvA) oscillations are seen to onset above approximately 20 T. At low angles ($\leq 35°$) a distinct peak appears before a gradual decay while at high angles ($\geq 40°$) a broad shoulder covers the whole field range, as exemplified by 15° and 60° data shown in main text Fig.1(c). This behavior is also observed in low field capacitive torque experiments performed in a superconducting magnet (see main text Fig. 4).

With increasing H, we identify distinct regimes for the observed overall torque response of Fe_3Sn_2. At low field (< 1 T) the magnetization evolves sharply, consistent with motion of soft ferromagnetic domains to be polarized along the field direction and $\tau \sim H^2$; as described in the main text the moment is aligned to better than 0.12 μ_B/f.u. by 2 T. This is followed by an approximately flat torque response up to 10 T, corresponding to further alignment of the moments along H to within 0.01 μ_B/f.u. Above this field scale we see the onset of dHvA oscillations and

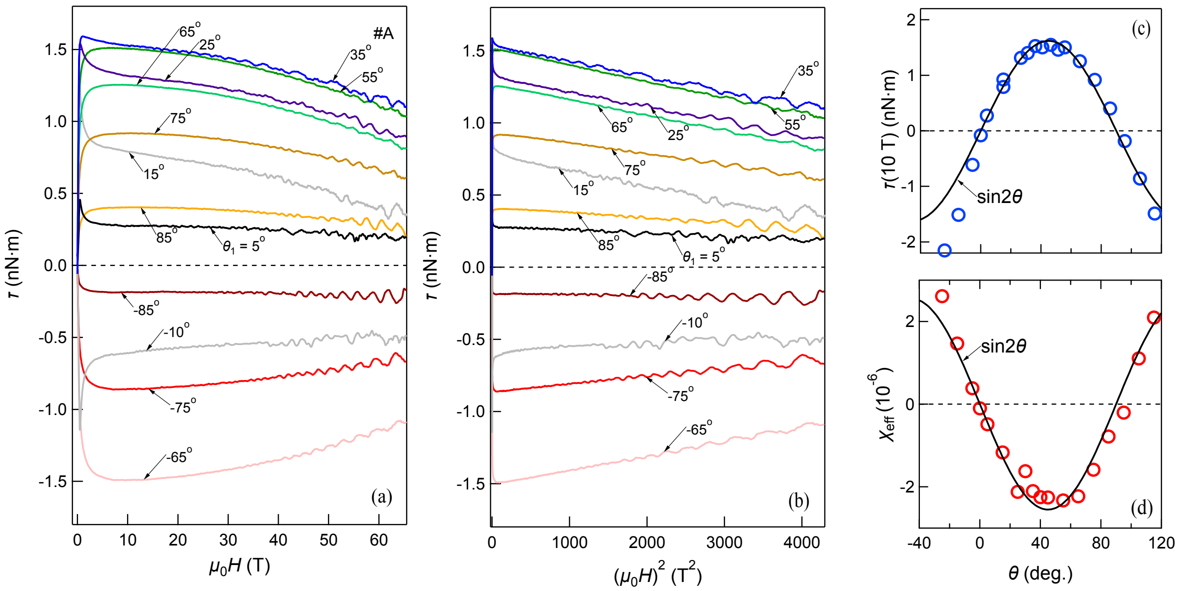

Supplementary Figure 1 | Torque response over extended angular range (a) The field profiles up to 65 T at base temperature of torque signal τ in Fe_3Sn_2 at selected angles for sample A with θ_1 rotation. (b) τ plotted against $(\mu_0 H)^2$. (c) Angular dependence of τ at 10 T. (d) Coefficient for the quadratic dependence χ_{eff} from panel (b) in the unit of volume susceptibility.

the appearance of a torque of opposite sign to that at low field (this appears as the small deviation from H^{-1} for $M_{\mathrm{T}}(H)$ in main text Fig. 4(b)). Plotting the torque response versus H^2 makes it clear that this high field deviation corresponds to a linear susceptibility. This is naturally associated with the electronic susceptibility that itself is the source of the dHvA oscillations. Here for this contribution to torque τ_1 we define an effective volume susceptibility χ_{eff} as the coefficient in τ linear in $(\mu_0 H)^2$ where $|\tau_1| = \chi_{\mathrm{eff}}(\mu_0 H)^2$. Here $\tau_1 = \mathbf{M} \times \mu_0 \mathbf{H} = \mu_0 (M_{ab} H_c - M_c H_{ab}) = \mu_0{}^2 (\chi_{ab} H_{ab} H_c - \chi_c H_c H_{ab}) = (\mu_0 H)^2 (\chi_{ab} \sin\theta \cos\theta - \chi_c \cos\theta \sin\theta) = (\mu_0 H)^2 (\chi_{ab} - \chi_c) \times \sin 2\theta / 2$, which gives $\chi_{\mathrm{eff}} = (\chi_{ab} - \chi_c) \sin 2\theta / 2$. This angular dependence is seen in Supplementary Fig. 1(d) and the resulting $\Delta\chi = \chi_{ab} - \chi_c \sim 4 \times 10^{-6}$ is on the same order of Pauli paramagnetism/Landau diamagnetism expected for a free electron system with comparable carrier density with Fe_3Sn_2 [1].

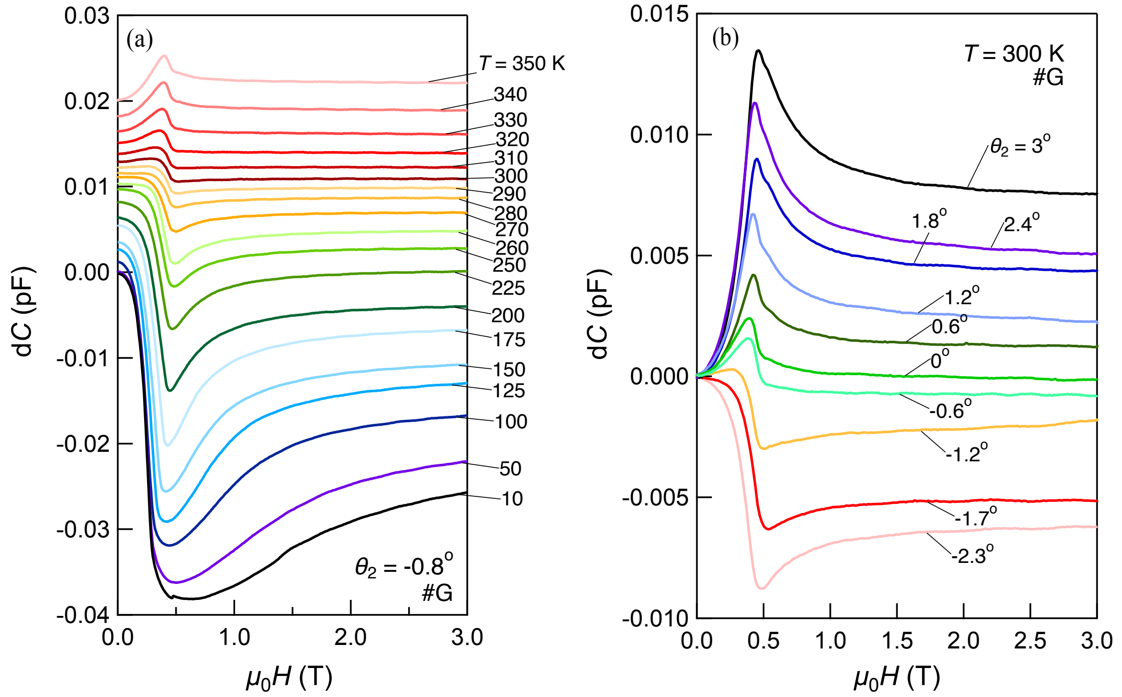

Supplementary Figure 2 | Capacitive torque response over extended temperature range (a) Torque response for Fe_3Sn_2 from 0-3 T with H aligned at -0.8° from the c-axis (traces are offset for clarity). (b) Torque response at selected angles close to c axis at room temperature.

We estimate the anisotropy energy K_1 using the angular dependent torque response at 10 T (Supplementary Fig. 1(c)) assuming the planar type anisotropy to the first order $E = K_1 \sin^2 \theta$. The corresponding torque response should be of the form $\tau = -\frac{\partial E}{\partial \theta} = -K_1 \sin 2\theta$. This gives us

$K_1 = -0.3 \times 10^5 \mathrm{J/m^3}$ for sample A which may include a considerable sample-dependent shape anisotropy in addition to the easy-plane type ferromagnetism at low temperatures.

In Supplementary Fig. 2(a) we show the low field torque response at an extended T range measured in a superconducting magnet. No hysteresis is observed. The sign change of the torque with T is a further indication of the bulk spin-reorientation [2, 3]. At high T, the torque profiles close to the easy c axis (Supplementary Fig. 2(b)) resembles that observed in uniaxial antiferromagnetic systems with H close to the Ising axis where the peak location concurs with the spin-flop field [4]. This suggests a potentially complex evolution of magnetism at low fields particularly at elevated T. An interesting direction for further study is to investigate the energetics of the massive Dirac and neighboring bands and their evolution in this process, as this may be a key to understanding the low energy scale for these changes.

Supplementary Note 2 | Angular dependence of the dHvA oscillations

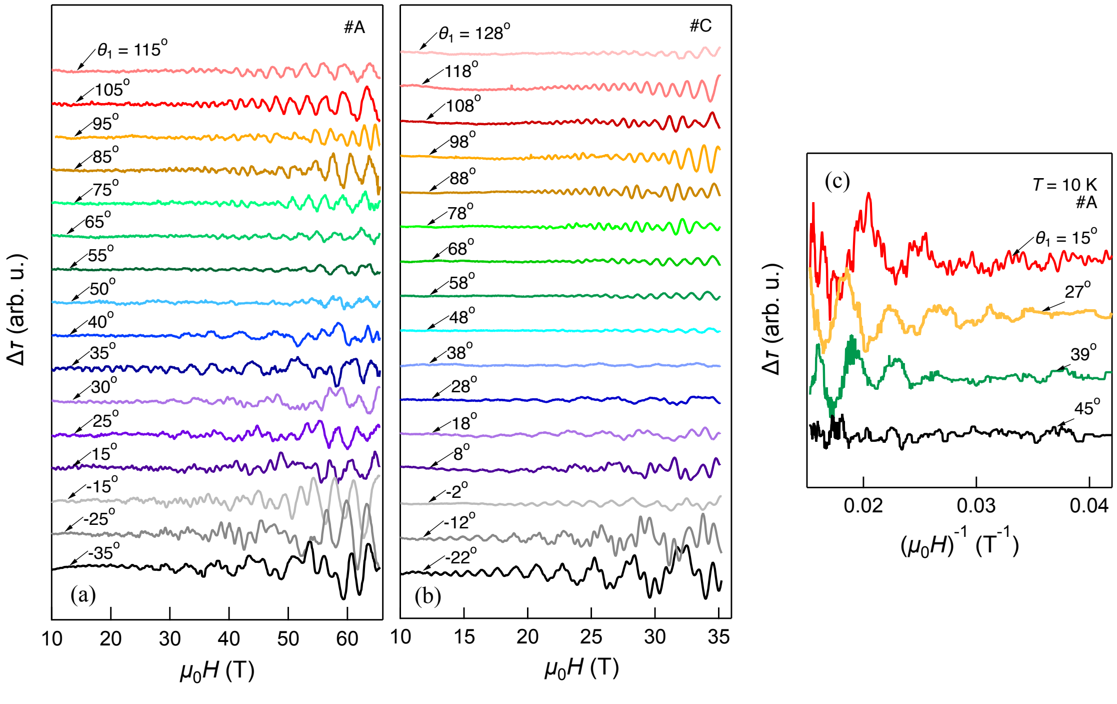

Supplementary Figure 3 | Torque dHvA oscillations (a)(b) The oscillatory part of the torque signal for sample A (a) and C (b), respectively, at the base temperature. (c) The oscillatory part of torque of sample A at selected angles at $T = 10$ K.

In Supplementary Fig. 3 we show the oscillatory part of the magnetic torque of sample A measured at $T = 0.53$ - 0.76 K at the pulsed facility in (a), of sample C measured at $T = 0.53$ - 0.76

K, DC field facilities in (b), respectively. In both sample A and C, at low angles close to the c-axis, there appears to be a dominating slow oscillation superimposed with series of faster frequencies. This slow series of oscillations persists to elevated temperature $T = 10$ K as shown in Supplementary Fig. 3(c). At high angles close to in-plane directions, a beating pattern between moderately fast oscillations can be seen in both A and C, as shown as the top curves in (a) and (b).

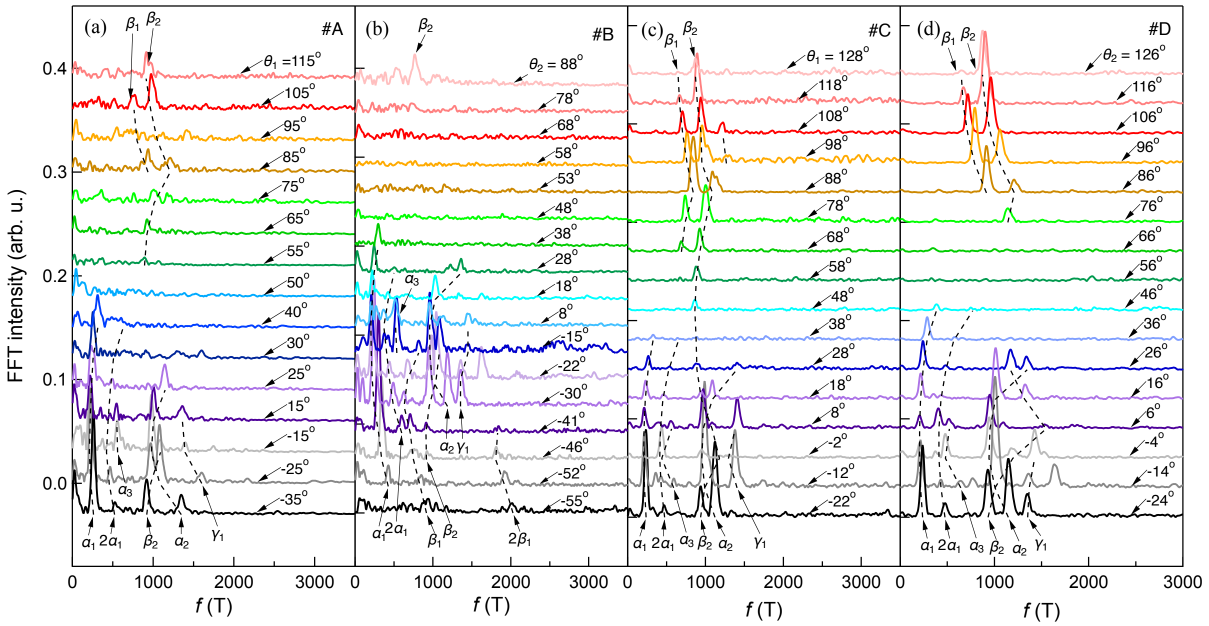

Supplementary Figure 4 | Fourier analysis of the dHvA oscillations (a-d) The Fast Fourier transform spectrum at a full angular range for sample A, B, C, D, respectively.

These behaviors are also reflected in the corresponding base temperature Fast Fourier transform (FFT) spectrums as shown in Supplementary Fig. 4. Here sample A and B are measured in the pulsed field facility and C and D are measured in the DC field facility, which show consistent FFT spectrums. At low angles close to the *c*-axis a rich series of oscillations are resolved where a weak branch of the second harmonic of α_1 is also visible. The frequencies of α_1 and α_2 grow quickly with increasing angle and are hardly resolved at angles above 50°, implying a quasi-2D nature of these Fermi pockets; the onset of this trend becomes clear above 35°, and is well captured by the two dimensional (2D) massive Dirac model described in the main text. At field orientations close to the kagome plane, the FFT spectra is composed of two peaks (a weak third frequency is also resolved at some angles) that are maximized at the in-plane direction, and we assign these to β_1 and β_2.

Supplementary Note 3 | Comparison of torque response in different in-plane directions

In sample A we have measured the torque response with rotation of *H* from *c* towards the two inequivalent in-plane directions (θ_1 and θ_2 rotations as defined in the main text Fig. 1(b)). The results are shown in Supplementary Figs. 5(a) and (b), respectively. The behaviors are similar in

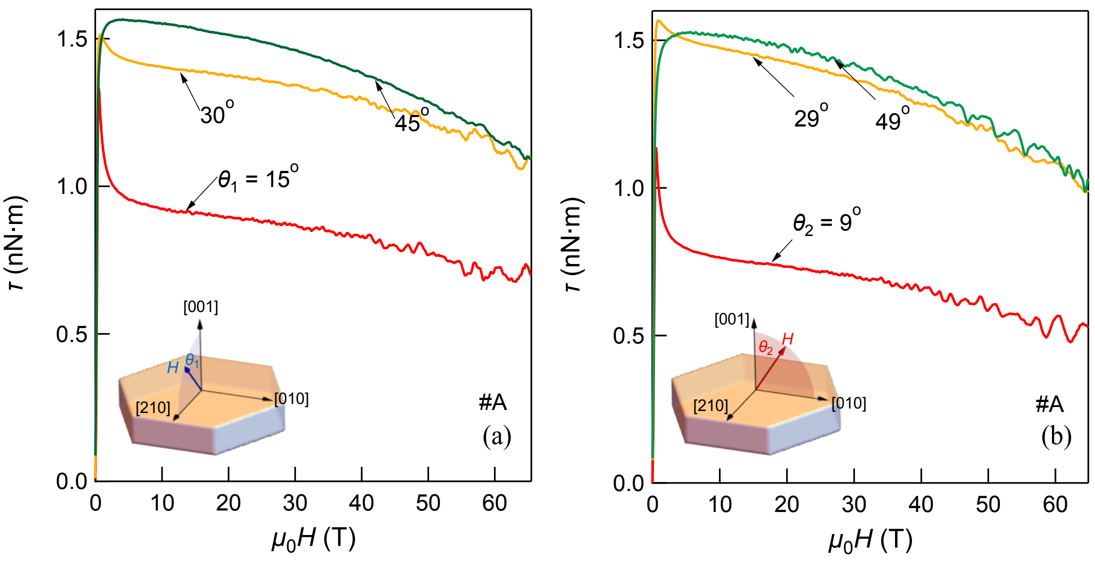

Supplementary Figure 5 | Comparison between θ_1 and θ_2 rotations Torque response at selected angles for rotation from [001] to the two inequivalent in-plane directions [210] and [010] are shown in (a) and (b), respectively.

both the overall torque and dHvA response (for the frequency behaviors see Fig. 2(a)), suggesting the system is relatively isotropic in the kagome plane, though experiments directly probing the response as a function of in-plane field rotation are of significant interest.

Supplementary Note 4 | Anisotropic magnetoresistance in Fe_3Sn_2

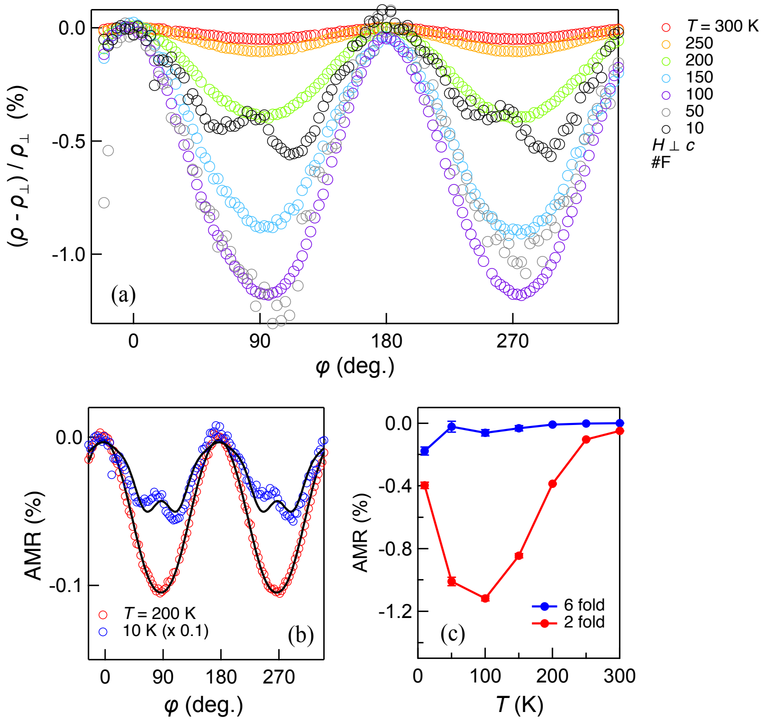

Supplementary Figure 6 | Anisotropic magnetoresistance in Fe_3Sn_2 (a) Anisotropic magnetoresistance (AMR) in Fe_3Sn_2 at 3 T at selected temperatures. (b) Fitting of the AMR responses using polynomials of $\cos\theta$ at T = 200 K and 10 K. (c) The coefficient of 2-fold and 6-fold behavior of AMR.

We show the anisotropic magnetoresistance (AMR) in Fe_3Sn_2 with rotating magnetic field within the kagome plane and current flowing along the [010] direction in Supplementary Fig. 6. We define $\rho_\perp(\rho_{||})$ as the resistivity when magnetic field is applied perpendicular (parallel) to the current and along the [210] ([010]) direction. At all T the negative sign of AMR ratio $(\rho_{||} - \rho_\perp)/\rho_\perp$ is opposite with that typically observed in elemental ferromagnetic metals Fe, Co and Ni [5]. At low temperature near 10 K a weak six-fold in-plane anisotropy starts to develop. We use the following formula to describe the AMR behavior observed here:

$$\mathrm{MR} = (A_1 + A_2 \cos^2 3\theta) \cos^2\theta + A_0 \qquad (1)$$

Where A_1 describes the two-fold anisotropy defined by the current and A_2 describes the six-fold anisotropy related to the in-plane ferromagnetic order. Typical fitting curves to Supplementary Eqn. 1 can be seen in Supplementary Fig. 6(b) and the temperature dependence of the fitting parameters are shown in Supplementary Fig. 6(c) where the two-fold symmetry defined by the current direction is dominating over a wide temperature range and comparable with the six-fold symmetry at the lowest temperature.

Supplementary Note 5 | Temperature/Field dependences of dHvA oscillations

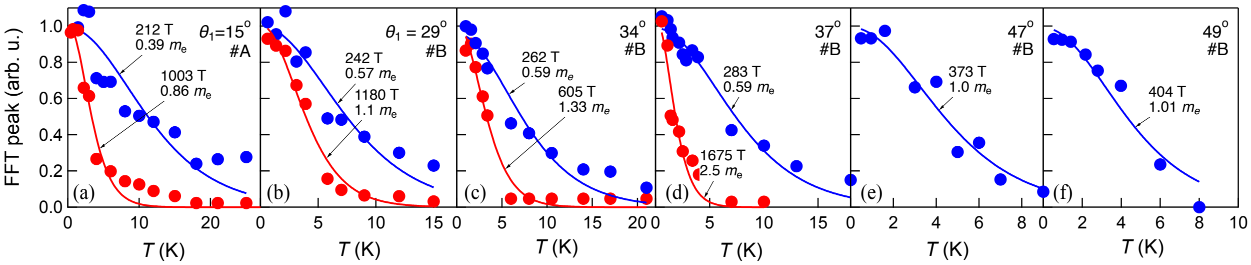

Supplementary Figure 7 | Temperature dependence of dHvA oscillations Lifshitz-Kosevich fitting to extract the effective mass at selected angles. Blue represents the α_1 oscillations and red represents α_2 oscillations. The α_2 oscillations are not resolved at and above 47°.

In Supplementary Fig. 7 we show the Lifshitz-Kosevich (LK) fitting for the normalized FFT peak intensities at various angles (see main text). The blue circles correspond to α_1 oscillations and red circles to α_2 oscillations.

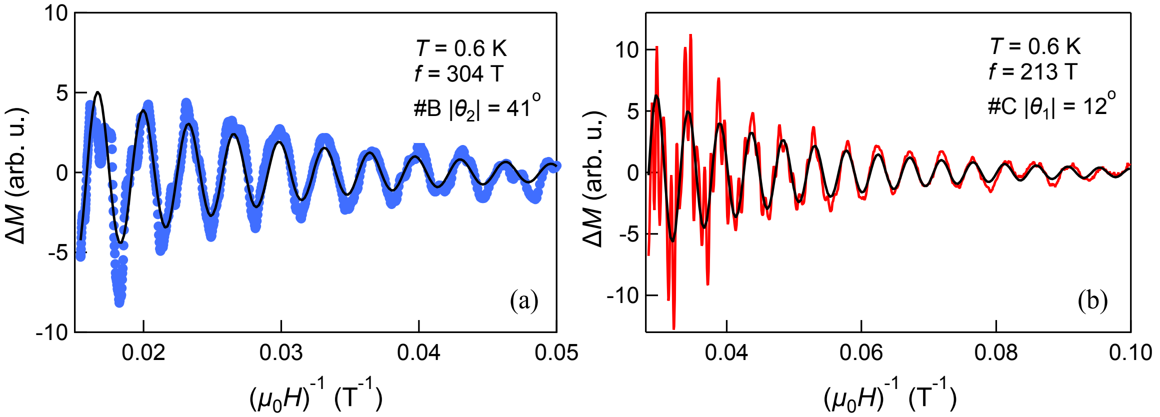

Supplementary Figure 8 | LK fitting of the oscillatory pattern Oscillatory magnetization (blue circles) and fit to the field profile of the α_1 oscillation (Eqn. 1 main text) for sample B (panel (a)) and sample C (panel (b)).

Supplementary Fig. 8 shows the field dependence of the oscillation amplitude from which we may extract the Dingle temperature T_D. For sample B (Supplementary Fig. 8(a)) we find $T_D = (5.6 \pm 0.6)$ K corresponding to a quantum scattering time τ_Q of 2.2 $\times 10^{-13}$ s via $T_D = \hbar/2\pi k_B \tau_Q$, and a mean free path of ~ 50 nm given $v_F^0 = 2.2 \times 10^5$ m s^{-1}. The estimated mobility for α_1 is around ~ 800 cm^{2}V^{-1}s^{-1}, consistent with the observation that at lowest T the quantum oscillations emerge near 20 T. Other samples show similar values of T_D: (8.8 ± 0.9) K for sample A,

(4.6 ± 0.8) K for sample C, and (4.9 ± 0.6) K for D with a fit shown in Supplementary Fig. 8(b) where oscillations are seen to onset at approximately 10 T.

Supplementary Note 6 | Comparison with the Yamaji model

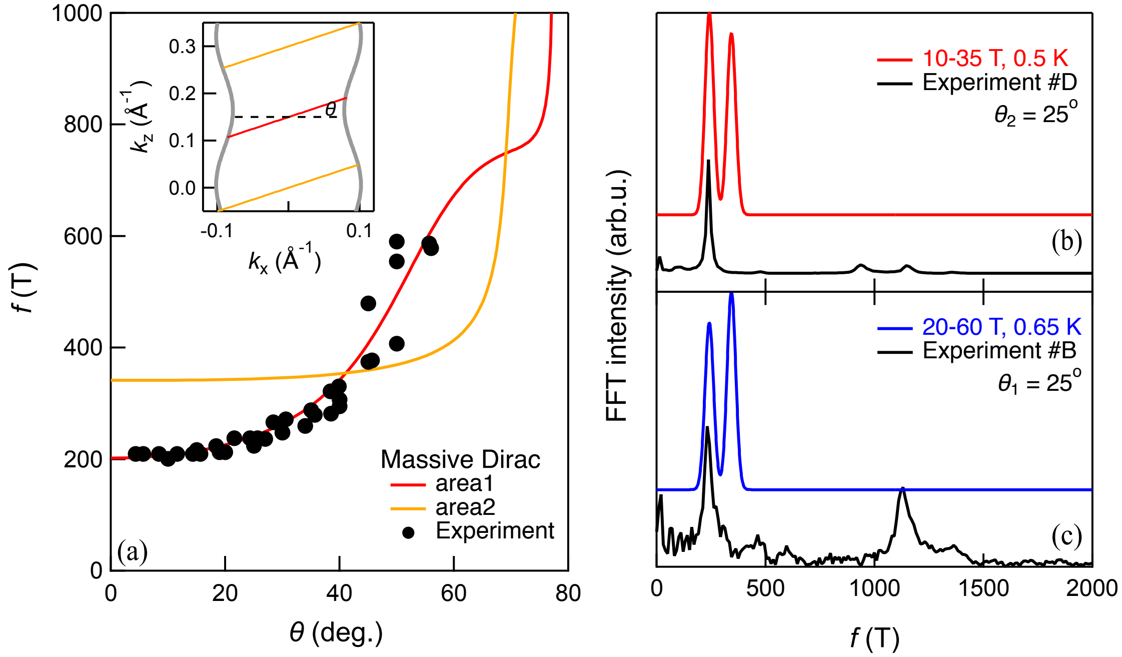

Supplementary Figure 9 | Comparing the Yamaji model with experiments (a) Simulated quantum oscillation frequencies with an in-plane massive Dirac model and an out of plane sinusoidal k_z dispersion with parameters described in text. The red and orange lines are simulations for the frequencies of the neck and belly orbits of the Fermi surface as shown in inset. (b)(c) Simulated FFT spectrum for given T and H ranges compared with DC (b) and pulsed field (c) results at $\theta = 25°$, where the lowest frequency oscillation is most prominently observed.

An alternative scenario to describe the faster than $1/\cos\theta$ evolution of the observed α frequencies is a quasi-2D Fermi surface with k_z-warping. We construct here for comparison a simple Yamaji model with a sinusoidal warping for in-plane massive Dirac dispersions [6]. The Hamiltonian takes the form $\mathcal{H} = t\cos(k_z c) + \sqrt{(\frac{\Delta}{2})^2 + (\hbar v_\mathrm{D} k_x)^2}$. where k_x represents the in-plane Fermi wavevector at each k_z, the vertical lattice constant c is taken to be 20 Å, and t is the out-of-plane transfer integral. With $v_\mathrm{D} = 2.2 \times 10^5$m/s, $\Delta = 20$ meV, $t = 17$ meV, and Fermi

energy $E_F = 131$ meV we get an angular evolution of the two extremal orbits as shown in Supplementary Fig. 9(a) with the oscillation originating from the neck orbits (red curve) comparable with the experimental observations. In this context, a partner belly frequency (see Supplementary Fig. 9(a) inset) with relatively weak angular dispersion in our measured angular range is expected which intersects the neck branch at Yamaji angles 41° and 69°. Moreover, focusing on low angles, the oscillation amplitudes of both frequencies are proportional to $\sim R_D R_T / (\partial^2 A / \partial k_{||}^2)$ [7] (here $k_{||}$ represents the wave vector parallel to H and A is the Fermi surface cross section perpendicular to H at a given $k_{||}$, and $\partial^2 A / \partial k_{||}^2$ is inversely proportional to the density of states at a given Fermi surface cross section). We therefore expect comparable FFT amplitudes for the belly and neck frequencies (simulated for $\theta = 25°$ as red and blue curves in Supplementary Figs. 9(b)(c)) which is inconsistent with our observations (black curves in Supplementary Figs. 9(b)(c)). We note that in the above model a range of band parameters can be used to capture the low angle frequency for α_1, but generally predict comparable partner frequencies and Yamaji angles.

Supplementary Note 7 | Tight-binding model of the bilayer kagome lattice

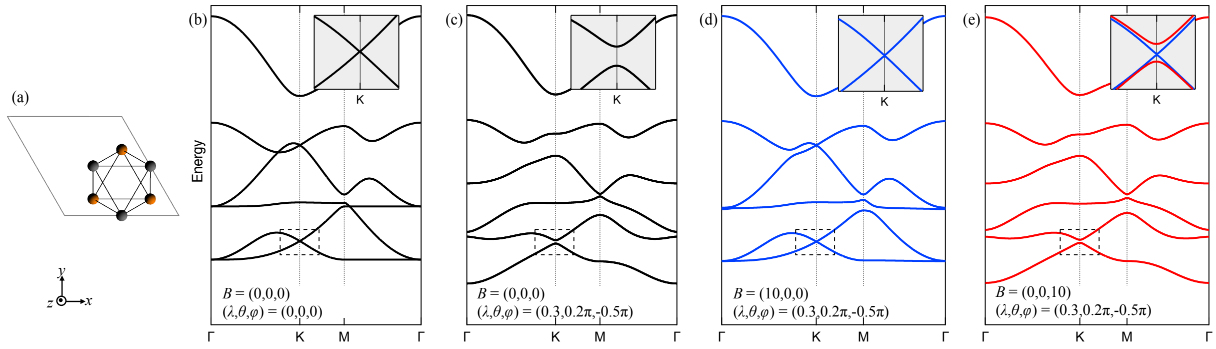

Supplementary Figure 10 | Tight-binding model of the bilayer kagome lattice (a) Schematic of the bilayer kagome lattice in Fe_3Sn_2 where gray and orange atoms reside on different layers. A typical set of dispersions are shown in (b-e) (parameters see text): (b) time-reversal symmetric (TRS) case without spin-orbit coupling (SOC), (c) TRS case with SOC, (d)(e) with in-plane ferromagnetic order and out-of-plane ferromagnetic order and SOC. The insets of (b-d) panels show a magnified view highlighted by the dashed box in each panel and the inset in (e) compares the region of the Dirac crossing at K for both in-plane (blue) and out-of-plane (red) ferromagnetic order.

To theoretically shed light on the behavior of the ferromagnetic Dirac fermions, we construct a nearest neighbor tight-binding model of the bilayer kagome lattice (the fundamental network of Fe in Fe_3Sn_2) which includes ferromagnetism and spin-orbit coupling. The tight-binding Hamiltonian is constructed based on the *s* orbital and there are 3 atoms per layer and 2 spins per atom (↑, ↓) (as shown in Supplementary Fig. 10(a)):

$$H = \sum_{<ij>ab\sigma} t_{ab}(c^{\dagger}_{ia\sigma}c_{jb\sigma} + \gamma\delta_{ab}c^{\dagger}_{ja\sigma}c_{ib\sigma}) + i\lambda \sum_{<ij>a\alpha\beta} (\mathbf{E}_{ij} \times \mathbf{R}_{ij}) \cdot \sigma_{\alpha\beta}c^{\dagger}_{ia\alpha}c_{ja\beta} + \mathbf{B}_i \cdot \boldsymbol{\sigma}_i \quad (2)$$

Here in Supplementary Eqn. 2 the indices $(i, j) = (1, 2, 3)$ represent the different kagome sites within each layer and $<ij>$ represents the nearest hopping. $(a, b) = (1, 2)$ represent different layers and $(\alpha, \beta) = (\uparrow, \downarrow)$ represents the spin degree of freedom. t_{ab} is the nearest hopping parameter and δ is the Kronecker delta. Due to the lattice symmetry, $t_{11} = t_{22} = t$ and $t_{12} = t_{21} = t'$. We introduce an additional parameter γ to characterize the imperfection of kagome lattice within each plane ($\gamma = 1$ corresponds to the perfect kagome lattice) [8]. In the second sum in Supplementary Eqn. 2, λ is the strength of spin-orbital coupling, $\mathbf{E}_{ij}$ is the local electric field in the hopping path along $\mathbf{R}_{ij}$ direction. $\mathbf{B}_i$ is the local magnetic field, which we employ to simulate the effect of ferromagnetic order along different directions. We use θ and ϕ to represent the direction of the electric field: θ is the polar angle defined away from *z* axis (as shown in Supplementary Fig. 10(a)) and ϕ is the azimuth angle in the plane defined away from each in-plane nearest neighbor bonding $\mathbf{R}_{ij}$. We note that on the bilayer kagome lattice both in and out-of-plane electric fields are symmetrically allowed.

The set of bands shown in Supplementary Fig. 10 are calculated using the following parameters: $t = t' = 1, \gamma = 1.01$ while the λ, θ, ϕ values are noted in each panel. Without spin-orbit coupling nor magnetic order the system shows a massless Dirac crossing at K (Supplementary Fig. 10(b)), which is protected by the crystallographic symmetries [8]. With the introduction of spin-orbit coupling a gap opens at this Dirac point (Supplementary Fig. 10(c)). When the Fermi level is placed within the gap this results in a time-reversal symmetric topological insulating phase. Further introducing the ferromagnetic order gives rise to a moment-orientation-dependent gap (Supplementary Figs. 10(d,e)) where the gap is opened exclusively when the moment is out-of-plane. This reflects the selection rule $\mathbf{s} \cdot (\mathbf{E}_{ij} \times \mathbf{R}_{ij})$ dedicated by the microscopic form of spin-orbit coupling [9]. We note that the present model only takes into account the lattice and the conclusions regarding band topology hold in a general sense. Further consideration of the orbital

degrees of freedom would potentially introduce change in the Fermi velocity and Fermi energy, as observed experimentally.

Supplementary References

[1] G. Grosso and G. P. Parravicini, *Solid State Physics*, *Second Edition*, Academic Press, (Oxford, United Kingdom, 2014).

[2] Z. Hou, W. Ren, B. Ding, G. Xu, Y. Wang, B. Yang, Q. Zhang, Y. Zhang, E. Liu, F. Xu, W. Wang, G. Wu, X. Zhang, B. Shen and Z. Zhang, Observation of various and spontaneous magnetic skyrmionic bubbles at room temperature in a frustrated kagome magnet with uniaxial magnetic Anisotropy, Adv. Mater. 29, 1701144 (2017).

[3] L. A. Fenner, A. A. Dee and A. S. Wills, Non-collinearity and spin frustration in the itinerant kagome ferromagnet Fe_3Sn_2, J. Phys.: Condens. Matter 21, 452202 (2009).

[4] M. Tokumoto, H. Tanaka, T. Otsuka, H. Kobayashi and A. Kobayashi, Observation of spin-flop transition in antiferromagnetic organic molecular conductors using AFM micro-cantilever, Polyhedron, 24, 2793-2795 (2005).

[5] S. Kokado, M. Tsunoda, K. Harigaya and A. Sakuma, Anisotropic Magnetoresistance Effects in Fe, Co, Ni, Fe_4N, and Half-Metallic Ferromagnet: A Systematic Analysis, J. Phys. Soc. Jpn. 81, 024705 (2012).

[6] K. Yamaji, On the Angle Dependence of the Magnetoresistance in Quasi-Two-Dimensional Organic Superconductors, J. Phys. Soc. Jpn. 58, 1520-1523 (1989).

[7] D. Shoenberg, *Magnetic Oscillations in Metals.* Cambridge University Press (Cambridge, United Kingdom, 1984).

[8] L. Ye *et al.*, Massive Dirac fermions in a ferromagnetic kagome metal. Nature 555, 638-642 (2018).

[9] C. L. Kane and E. J. Mele, Quantum Spin Hall Effect in Graphene, Phys. Rev. Lett. 95, 226801 (2005).
